# Successful treatment of rituximab-unresponsive elderly-onset neuromyelitis optica spectrum disorder and hypogammaglobulinemia with ofatumumab plus intravenous immunoglobulin therapy in a patient with mutant *FCGR3A* genotype: A case report

**DOI:** 10.3389/fimmu.2022.1047992

**Published:** 2022-12-08

**Authors:** Weihe Zhang, Yujuan Jiao, Jinsong Jiao, Ming Jin, Dantao Peng

**Affiliations:** ^1^ Department of Neurology, China-Japan Friendship Hospital, Beijing, China; ^2^ Department of Ophthalmology, China-Japan Friendship Hospital, Beijing, China

**Keywords:** neuromyelitis optica spectrum disorder, ofatumumab, rituximab, hypogammaglobulinemia, case report

## Abstract

**Background:**

Elderly-onset neuromyelitis optica spectrum disorder (NMOSD) is a rare entity that poses a therapeutic challenge. We report a case of elderly-onset NMOSD with mutant *FCGR3A* genotype who was successfully treated with ofatumumab after multiple episodes of relapse.

**Case Report:**

The patient was a 67-year-old woman who was diagnosed with NMOSD with high disease activity. She experienced six episodes of relapse over a period of 2 years despite immunosuppressant therapy with intravenous rituximab (RTX), oral steroids, mycophenolate mofetil, and tacrolimus. At the last relapse, she was unable to walk and developed immunosuppressant-induced hypogammaglobulinemia. Based on the insufficient B cell depletion and *FCGR3A-FF* genotype carrier, the patient was diagnosed as RTX non-responder. After subcutaneous ofatumumab plus intravenous immunoglobulin replacement therapy, she was able to walk independently, and experienced no further relapse. Ofatumumab was well-tolerated, and sufficiently depleted the circulating B cells.

**Conclusion:**

Ofatumumab might be an effective alternative in RTX-unresponsive NMOSD, and seems to be safe in elderly patients.

## Introduction

Neuromyelitis optica spectrum disorder (NMOSD) is an autoimmune disorder of the central nervous system that typically occurs in young women. Elderly-onset NMOSD is a rare entity, but is associated with the same relapse frequency and more severe attacks (1). The therapeutic decision-making is more challenging in elderly patients due to multiple comorbidities and high risk of drug-induced side effects. Ofatumumab is the first fully human anti-CD20 monoclonal antibody which has been approved for relapsing forms of multiple sclerosis. However, the efficacy of ofatumumab against NMOSD is unclear. Recently, ofatumumab was reported to be effective in a pediatric patient with NMOSD who failed to respond to rituximab (RTX) (2). Herein, we describe a case of RTX-unresponsive elderly-onset NMOSD with mutant *FCGR3A* genotype that also showed favorable response to ofatumumab. Moreover, the immunosuppressant-induced hypogammaglobulinemia posed a therapeutic challenge in this patient.

## Case presentation

**Figure 1:**
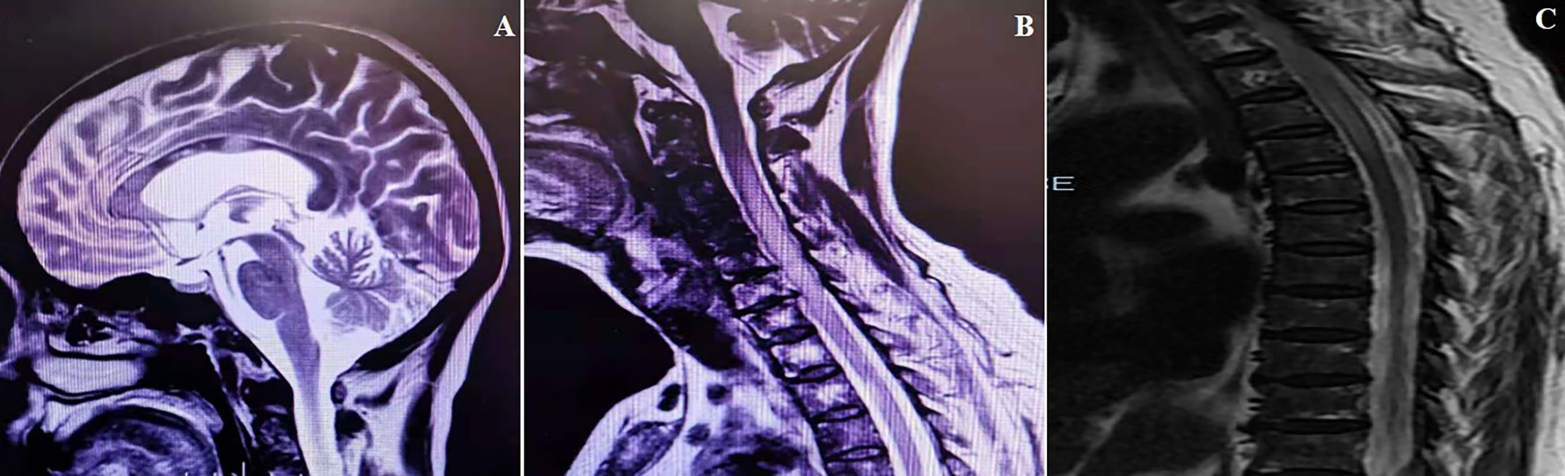
MRI scans obtained at the time of the first attack. **(A)** Cerebral MRI (sagittal T2 image) exhibiting disseminated lesions in corpus callosum. **(B, C)** Spinal MRI (sagittal T2 image) exhibiting longitudinally extensive lesion from C3 to T5.

**Figure 2:**
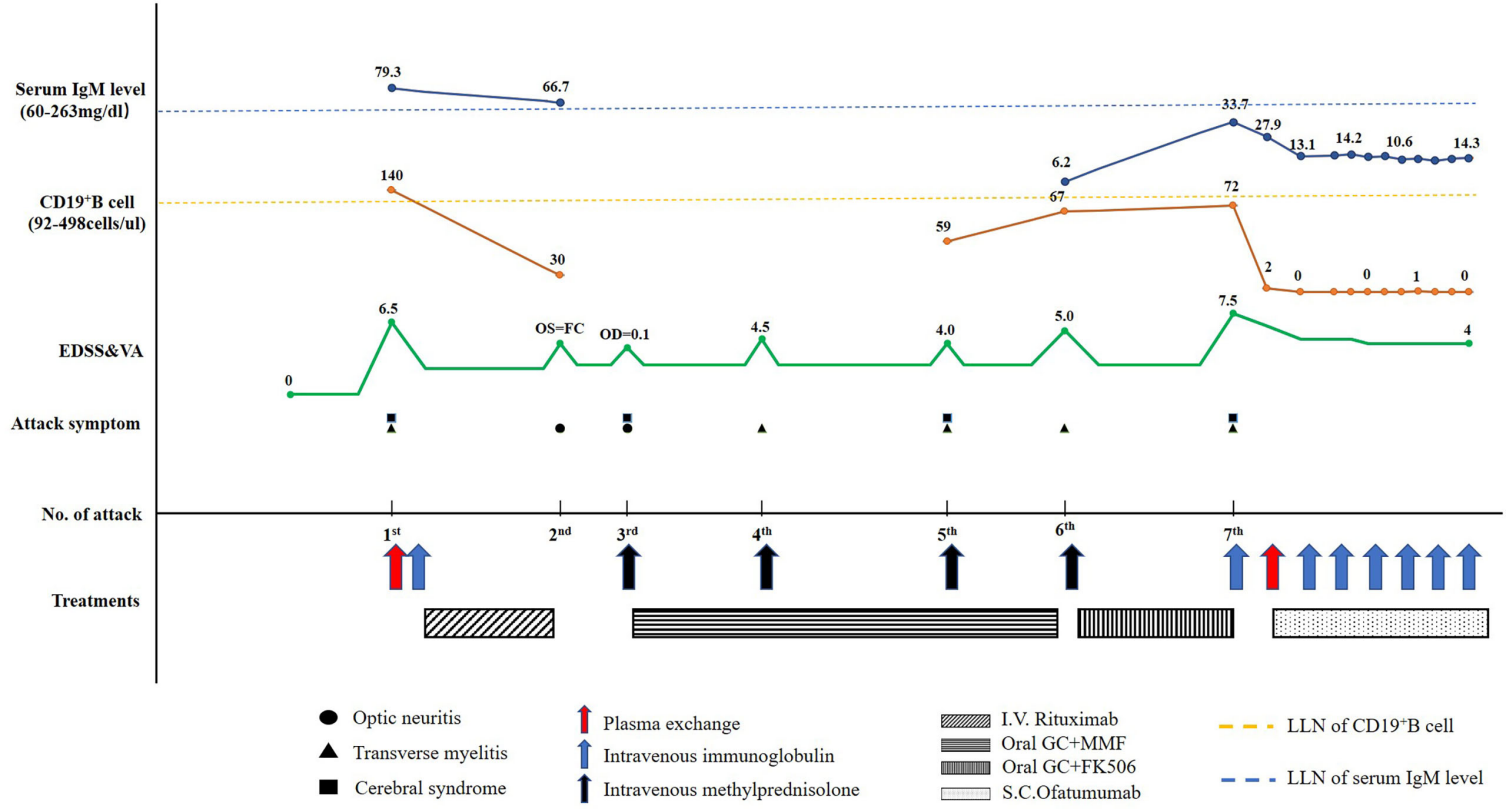
Schematic illustration of the disease course showing the temporal sequence of symptoms, disability, treatment details, CD19^+^B cell count, and serum IgM level. EDSS, Expanded Disability Status Scale; FC, finger count; FK506, tacrolimus; GC, glucocorticoids; I.V., intravenous; S.C., subcutaneous; MMF, mycophenolate mofetil; OD, right visual acuity; OS, left visual acuity; LLN, lower limits of normal; VA, visual acuity.

A 67-year-old woman was admitted to the China-Japan Friendship Hospital (Beijing, China) due to bilateral needle-like pain in upper back, lower extremity weakness, and sphincter dysfunction for one month. She had undergone thyroid cancer surgery four years ago, and was treated with oral levothyroxine sodium (Euthyrox^®^, 75 μg per day) to maintain normal thyroid function. She was also diagnosed with Sjogren Syndrome, but had not received any immunosuppressive treatment prior to admission. On admission, she exhibited malaise and was not able to walk; muscle strength in the right and left lower extremity was graded as 2/5 and 4/5 (Medical Research Council), respectively. There was bilateral tendon hyperreflexia and positive Babinski sign. In addition, she had hypoesthesia at the level of 6th thoracic segment. The Expanded Disability Status Scale (EDSS) score at nadir was 6.5. The diagnosis of NMOSD was established based on seropositivity for AQP4-IgG and longitudinally extensive transverse myelitis (LETM, continuous spinal cord lesions extending from cervical to thoracic segments) (**Figure 1**). No other abnormalities were detected on additional specific tests for autoimmune disorders (serum myelin oligodendrocyte glycoprotein antibodies and glial fibrillary acidic protein antibodies), metagenomic next-generation sequencing of cerebrospinal fluid for microbial infection, hematological examination, serum angiotensin-converting enzyme, tumor markers, and immunoglobulin levels. After 5 cycles of plasma exchange and 5 infusions of intravenous immunoglobulin (IVIg), the patient showed marked alleviation of symptoms and regained the ability to walk (EDSS=3.5). RTX standardized induction protocol (375 mg/m^2^ infused once weekly for 4 weeks) was used to prevent relapse. Unfortunately, two months after RTX induction therapy, she suffered another severe attack with left optic neuritis (visual acuity=finger count) with insufficient depletion of CD19^+^B cells (**Figure 2**). Based on the *FCGR3A*-*FF* genotype carrier, we believed that the patient was RTX non-responder. Sanger sequencing to determine *FCGR3A-V158F (rs396991)* gene polymorphism was performed by an independent medical agency. Therefore, we decided to switch the therapeutic strategy. However, she experienced five additional episodes of relapse during the last 1.5 years, despite sufficient immunosuppression with oral steroids (prednisone, 10–60 mg per day), mycophenolate mofetil (1000 mg, twice daily), and tacrolimus (1.5 mg, twice daily) (**Figure 2**). After the last relapse, she was severely disabled (bilateral optic atrophy, EDSS=7.5) and developed immunosuppressant-induced hypogammaglobulinemia (with greatest impact on IgM). In consideration of the first-line treatment resistance and secondary immunodeficiency, subcutaneous ofatumumab was prescribed: once weekly injection of 20 mg for 3 weeks and then one injection of 20 mg every 4 weeks, in combination with IVIg (2 g/kg each month for the first 3 months followed by 1.2 g/kg each month) replacement therapy. Six months later, she was able to walk independently (EDSS=4.0), and experienced no further relapse. The peripheral CD19^+^B cell count decreased to 2 cells/μL (reference range, 92–498 cells/μL) after the first dose and was maintained at low-level during ofatumumab treatment. Serum IgM level remained stable (**Figure 2**).

## Discussion

As the first-generation anti-CD20 antibody, RTX depletes B cells mainly *via* antibody-dependent cell-mediated cytotoxicity pathways due to the linking of fragment c gamma receptors (FcγR IIIA) on natural killer cells (3). Consequently, patients with *FCGR3A-V158F* genetic mutation may have greater probability of insufficient depletion of B cells, and relapse during RTX treatment (4). Ofatumumab exhibits a greater potency in recruiting complement than RTX, thus exerting a higher complement-dependent cytotoxic efficacy (5). Thus, the low FcγR IIIA pathway dependent property of ofatumumab may explain its better efficacy than RTX in the present case, even in the presence of homozygous mutation of *FCGR3A-V158F* genotype.

In addition, hypogammaglobulinemia may occur during prolonged treatment with anti-CD20-depleting therapies and may lead to serious infections. In randomized controlled trials, a small percentage of ofatumumab-treated patients developed decreased immunoglobulin levels (6, 7). Our patient developed drug-induced hypogammaglobulinemia, but still showed high disease activity. Therefore, subcutaneous ofatumumab plus IVIg replacement therapy were introduced. Fortunately, ofatumumab was well-tolerated by our patient. Besides, ofatumumab decreased the probability of relapse with sufficient depletion of B cells.

In summary, the current case suggests that ofatumumab might be an effective alternative in patients with incomplete B cell depletion after RTX genetic testing in NMOSD, and highlights the potential safety of ofatumumab in elderly patients.

## Data availability statement

The original contributions presented in the study are included in the article/supplementary material. Further inquiries can be directed to the corresponding authors.

## Ethics statement

The studies involving human participants were reviewed and approved by Institutional Review Board of China-Japan Friendship Hospital. The patients/participants provided their written informed consent to participate in this study.

## Author contributions

WHZ drafted the manuscript. WHZ, YJJ, JSJ, MJ, and DTP prepared the materials, collected, and analyzed the data. WHZ and DTP revised the manuscript. All authors contributed to the article and approved the submitted version.
